# Development and Structural Variety of the Chondroitin Sulfate Proteoglycans-Contained Extracellular Matrix in the Mouse Brain

**DOI:** 10.1155/2015/256389

**Published:** 2015-11-16

**Authors:** Noriko Horii-Hayashi, Takayo Sasagawa, Wataru Matsunaga, Mayumi Nishi

**Affiliations:** Department of Anatomy and Cell Biology, Nara Medical University, 840 Shijo-cho, Kashihara, Nara 634-8521, Japan

## Abstract

Chondroitin sulfate proteoglycans (CSPGs) are major components of the extracellular matrix (ECM) in the brain. In adult mammals, CSPGs form the specialized ECM structure perineuronal nets (PNNs) that surround somata and dendrites of certain types of neurons. PNNs restrict synaptic plasticity and regulate the closure of critical periods. Although previous studies have examined the starting period of PNN formation, focusing on primary sensory cortices, there are no systematic studies at the whole brain level. Here, we examined the starting period of PNN formation in male mice ranging in age from postnatal day 3 to week 11, mainly focusing on several cortical areas, limbic structures, hypothalamus, and brain stem, using lectin histochemistry with *Wisteria floribunda* agglutinin (WFA). Results showed that early PNN formation was observed in several reticular formations of the brain stem related to the cranial nerves and primary somatosensory cortices. In the limbic system, PNN formation in the hippocampus started earlier than that of the amygdala. Furthermore, in the medial amygdaloid nucleus and some hypothalamic regions, WFA labeling did not show typical PNN-like forms. The present study suggests spatiotemporal differences at the beginning of PNN formation and a structural variety of CSPG-contained ECM in the brain.

## 1. Introduction

Chondroitin sulfate proteoglycans (CSPGs) are major extracellular matrix (ECM) components in the central nervous system, and many types of CSPGs have been characterized in the brain [1]. Chondroitin sulfates (CS), glycosaminoglycan portions of CSPGs, are known as inhibitory substrates for neurite growth. CSPGs can act as axonal guidance molecules in the developing brain [2–4] and as stabilizing substrates for synapses in the adult brain [5, 6].

CSPGs are composed of a core protein and one or more CS glycosaminoglycans that covalently attach to the serine residues of the core protein. CS is composed of repeating disaccharide units of* N*-acetylgalactosamine and glucuronic acid. The lectin* Wisteria floribunda* agglutinin (WFA) is generally used to detect CS, owing to its ability to bind to the* N*-acetylgalactosamine within carbohydrate structures [7–9]. In the adult brain, WFA is known to label specialized ECM structures called perineuronal nets (PNNs), which are formed by aggregating multiple molecules, including CSPGs, hyaluronan, and glycoproteins. The majority of PNNs surround cell bodies and proximal dendrites of parvalbumin-positive inhibitory neurons in the cerebral cortex and limbic structures [7, 10, 11], whereas a few PNNs are found around pyramidal neurons in both the marsupial and primate cortex [12, 13].

Topographical distribution of CSPG changes during postnatal development, and the shape of PNNs gradually matures a few weeks after birth. Bahia et al. distinguished two successive ECM structures containing CSPGs in the developing barrel field of the primary somatosensory cortex (S1BF) in rats [14]: CSPG shows diffuse and neuropil-associated distribution in the first postnatal week. Next, CSPGs are concentrated around cell bodies with a PNN component structure at postnatal day 24 (P24). The shape of PNNs continues to develop and reach maturity by postnatal week 9 (9 w). A similar PNN structural development has been observed in the visual cortex [15].

Recent findings suggest the importance of PNNs for regulating synaptic plasticity and closing critical periods [15, 16]. However, the beginning periods of PNN formation in most brain regions other than the cortex have not been fully explored. If the appearance of PNNs implies the end of the critical period, we suspected that PNN appearance periods would be different among brain regions. This is because the maturation speed of each brain region is different depending on its functions. The present study investigated PNN formation in the mouse brain from P3 to 11 w using WFA staining, mainly focusing on the cortex, limbic system, hypothalamus, and brain stem. 

## 2. Materials and Methods

### 2.1. Animals

All experiments were performed using male C57BL/6N mice. All protocols followed NIH (USA) Guidelines and Guidelines for Proper Conduct of Animal Experiments published by the Science Council of Japan. Animals were purchased from Japan SLC, Inc. (Hamamatsu, Japan). The following number of animals was used at each stage: P3 (*n* = 8), P7 (*n* = 8), P9 (*n* = 4), P14 (*n* = 4), P21 (*n* = 4), 5 w (*n* = 4), and 11 w (*n* = 8). When using mice aged from P3 to P21, pregnant female mice at gestational day 14 were purchased from the same company and their pups were sampled at each stage; day of birth was considered P0. Mice were housed and maintained under standard laboratory conditions: 23°C and 55% humidity in a room with a 12 h light-dark cycle (lights on at 08:00 and off at 20:00), and food and water were available* ad libitum*.

### 2.2. Fixation and Histochemistry

Fixation and histochemical protocols were conducted as previously described [17]. Briefly, mice were anesthetized with sodium pentobarbitone (100 mg/kg) and transcardially perfused with heparinized 0.01 M phosphate-buffered saline (PBS, pH 7.4), followed by 4% paraformaldehyde in a 0.1 M phosphate buffer (pH = 7.4). Dissected brains were immersed with the same fixative either overnight or for 1 week; the former was applied to animals older than P14 and the latter to those from P3 to P9. Tissue sections were made using a cryostat (Leica, Wetzlar, Germany) at a thickness of 30 *μ*m. Free-floating sections were pretreated with 0.1% H_2_O_2 _in PBS for 20 min, immersed with PBS containing 0.3% Triton X-100 (PBST) for 15 min, and then treated with the blocking solution of PBST containing 5% normal horse serum for 2 h. When digesting CS, sections were treated with chondroitinase ABC (ChABC; 0.1 U/mL, Sigma, St. Louis, MO, USA) in 50 mM Tris-HCl buffer containing 30 mM sodium acetate (pH = 8.0) for 3 h at 37°C before blocking. The same treatment was performed without using ChABC as experimental counterparts. Sections were incubated with biotinylated WFA (dilution 1 : 1000, Vector Laboratories, Burlingame, CA) overnight at 4°C and developed using a Vectastain ABC Kit (Vector Laboratories). Sections were mounted on glass slides, dehydrated in graded ethanols, and coverslipped with Entellan (Merck, Darmstadt, Germany). Observations were carried out with a BX-43 transillumination microscope equipped with a FX630 CCD camera, and images were captured using 4, 10, 20, and 40x objective lenses, as well as a 100x immersion lens (Olympus). For fluorescent labeling, all procedures up to blocking were the same as above except for skipping H_2_O_2 _treatment. Sections were incubated with biotinylated WFA (dilution 1 : 500) at 4°C overnight and immersed with a mixture of Alexa 488-conjugated streptavidin (dilution 1 : 1000, Life Technologies) and NeuroTrace 530/615 (dilution 1 : 200, Life Technologies) for 2 h. Sections were mounted on slides and coverslipped with Vectashield containing 4′,6-diamidino-2-phenylindole dihydrochloride (DAPI; Vector Laboratories). Fluorescent images were captured by a FluoView 1000 confocal microscope in single-plane mode with a 40x objective lens (Olympus).

### 2.3. Regional Definitions

Brain regions were determined according to the mouse brain atlas [18] and the developing mouse brain atlas [19].

## 3. Results

The specificity of WFA for CS was confirmed by treating sections with ChABC, a CS digesting enzyme. Our results showed that ChABC treatment almost completely abolished WFA reactivity in all brain regions analyzed (see Figures S1A–G of the Supplementary Material available online at http://dx.doi.org/10.1155/2015/256389). However, in neonatal brain sections, especially at P3 (data not shown) and P7 (Figure S1H), small dot-like WFA reactivity was observed in both ChABC-treated and untreated sections. We considered this dot-like reactivity a false positive and excluded it from the CS-specific reaction.

**Figure 1:**
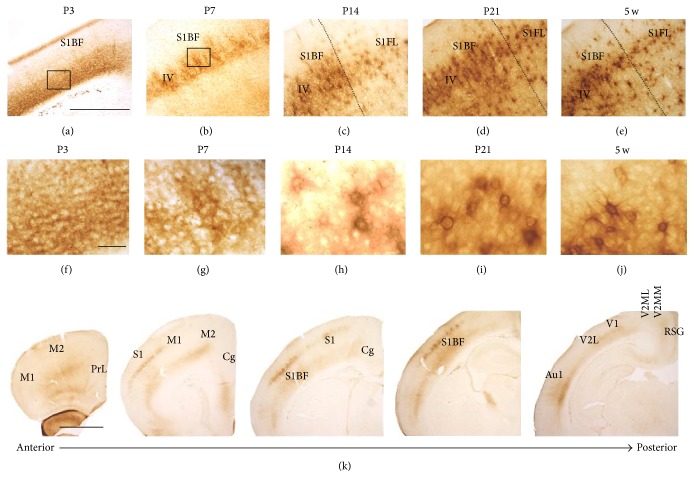
WFA-stained ECM in the developing cortex. (a–j) Low- (a–e) and high- (f–j) power images of WFA staining at P3 (a, f), P7 (b, g), P14 (c, h), P21 (d, i), and 5 w (e, j). Rectangular areas in (a) and (b) indicate the magnified areas shown in (f) and (g), respectively. High-power images (g–j) obtained from cortical IV layer of the S1BF. Dotted lines indicate the border between the S1BF and the S1FL. Accumulation of WFA reactivity around particular cell bodies was observed from P7 onward. WFA reactivity clearly surrounding both cell bodies and dendrites was observed from 5 w onward. (k) WFA-labeled serial coronal sections at P7, indicating that PNN-like WFA reactivity was observed in the S1BF, whereas any other regions indicated did not show PNN-like staining. Au1: primary auditory cortex; Cg: cingulate cortex; M1: primary motor cortex; M2: secondary motor cortex; PrL: prelimbic cortex; RSG: retrosplenial granular cortex; S1: primary sensory cortex; S1BF: barrel field of the primary somatosensory cortex; S1FL: forelimb primary somatosensory cortex; V1: primary visual cortex; V2L: lateral area of the secondary visual cortex; V2ML: mediolateral area of the secondary visual cortex; V2MM: mediomedial area of the secondary visual cortex. Scale bars = 500 (a–e), 50 (f–j), and 1000 (k) *μ*m.

Diffuse WFA reactivity was observed in the ventral layers of the S1BF at P3 (Figures 1(a) and 1(f)), and this observation was similar to a previous report [14]. At P7, WFA reactivity was observed in cortical IV layer (Figure 1(b)) and slightly condensed around particular cell bodies (Figure 1(g)), which were considered immature PNNs. At both P14 (Figures 1(c) and 1(h)) and P21 (Figures 1(d) and 1(i)), PNN-like labeling was observed around cell bodies without a clear dendritic surrounding. The typical PNN shape that is known to surround both cell bodies and dendrites was observed at 5 w (Figures 1(e) and 1(j)) and was almost equal to that seen at 11 w (data not shown). Serial WFA-labeled sections at P7 revealed that PNN-like labeling was observed in the primary somatosensory cortex (S1) and S1BF, whereas it could not be detected in the prelimbic (PrL), primary motor (M1), secondary motor (M2), cingulate (Cg), primary visual (V1), mediolateral (V2ML), and mediomedial (V2MM) areas of the secondary visual, primary auditory (Au1), and retrosplenial granular (RSG) cortices (Figure 1(k)). These results indicated an intercortical difference in the beginning of PNN formation.

**Figure 2:**
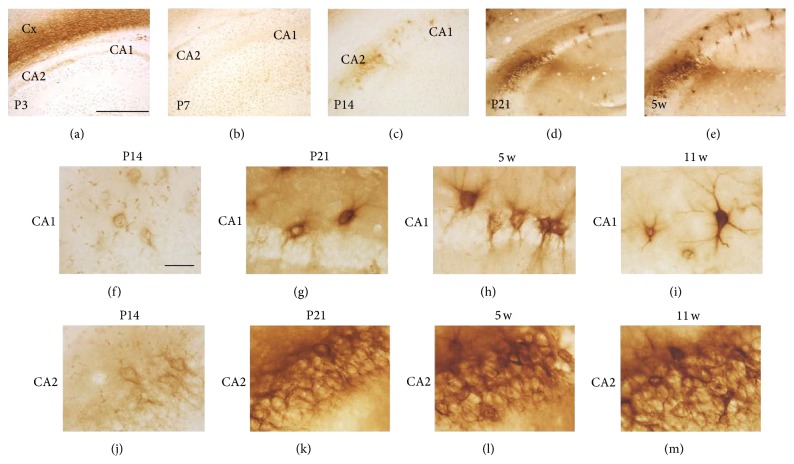
WFA-stained ECM in the developing hippocampus. (a–e) WFA-labeled images at P3 (a), P7 (B), P14 (c), P21 (d), and 5 w (e). PNN-like WFA reactivity was observed from P14 onward. (f–m) Higher magnification images of the CA1 (f–i) and CA2 (j–m) at P14 (f, j), P21 (g, k), 5 w (h, l), and 11 w (i, m). Diffuse PNN-like staining was observed in the CA1 at P14, which gradually surrounded cell bodies and dendrites from P21 onward. WFA reactivity in the CA2 from P21 onward was strong and showed a complex staining manner. Cx: cerebral cortex. Scale bars = 500 (a–e) and 50 (f–m) *μ*m.

In the hippocampal CA1, PNN-like WFA labeling could not be observed at P3 (Figure 2(a)) and P7 (Figure 2(b)), while it could be detected at P14 (Figure 2(c)). Similarly, clear WFA labeling was first observed in the hippocampal CA2 at P14 (Figure 2(c)). These labels became stronger and clearer at P21 (Figure 2(d)) and 5 w (Figure 2(e)). Higher magnification views confirmed PNN-like labeling in the CA1 at P14 (Figure 2(f)). From P21 onward (Figures 2(g)–2(i)), WFA reactivity clearly surrounded both cell bodies and dendrites in the CA1. WFA reactivity in the CA2 at P14 showed PNN-like labeling (Figure 2(j)), while, from P21 onward, the border of individual PNN structures was difficult to identify (Figures 2(k)–2(m)). Such a distinctive staining pattern in the CA2 is consistent with what has been previously reported [10, 20].

**Figure 3:**
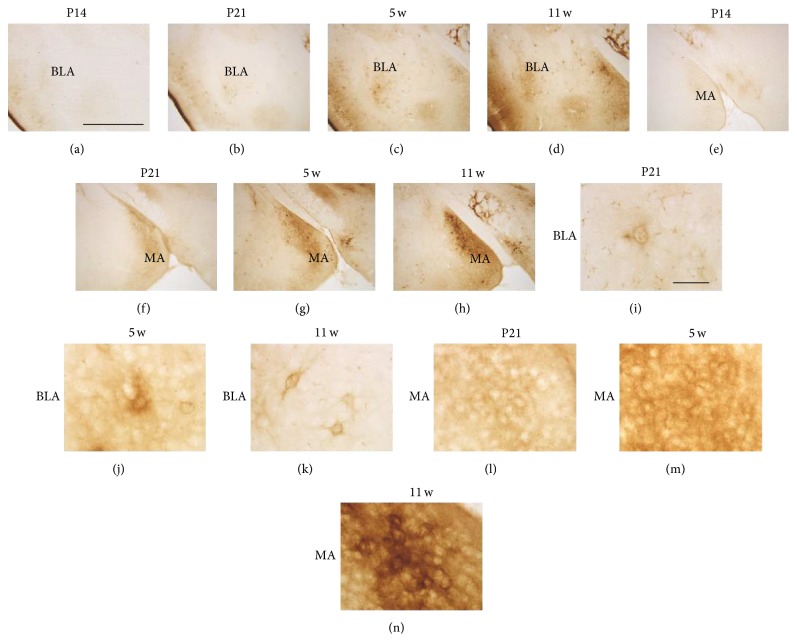
WFA-stained ECM in the developing amygdala. (a–h) WFA-labeled images in the BLA (a–d) and MA (e–h) at P14 (a, e), P21 (b, f), 5 w (c, g), and 11 w (d, h). In both regions, WFA reactivity was observed from P21 onward. (i–n) Higher magnification images of the BLA (i–k) and MA (l–n) at P21 (i, l), 5 w (j, m), and 11 w (k, n). Ambiguous PNN-like staining was observed in the BLA at P21 and 5 w, which surrounded both cell bodies and dendrites at 11 w. Diffuse neuropil-like staining was continuously observed in the MA at all stages shown and a loosely accumulated WFA reactivity around particular cell bodies was observed at 11 w. BLA: basolateral amygdaloid nucleus; MA: medial amygdaloid nucleus. Scale bars = 500 (a–h) and 50 (i–n) *μ*m.

In the amygdala, WFA reactivity was hardly observed by P14 in both the basolateral amygdaloid nucleus (BLA) (Figure 3(a)) and medial amygdaloid nucleus (MA) (Figure 3(e)). At P21, WFA reactivity in both subdivisions became detectable (Figures 3(b) and 3(f)). From 5 w onward, WFA reactivity was clearly observed in the BLA (Figures 3(c) and 3(d)) and MA (Figures 3(g) and 3(h)). High-power images further showed that an immature PNN-like form was observed in the BLA at P21 (Figure 3(i)), which gradually and clearly surrounded both cell bodies and dendrites from 5 w (Figure 3(j)) to 11 w (Figure 3(k)). However, in the MA, no clear WFA reactivity was observed to surround cell bodies and dendrites at P21 (Figure 3(l)) and 5 w (Figure 3(m)). At 11 w, WFA reactivity loosely accumulated around particular cell bodies but not around dendrites (Figure 3(n)).

**Figure 4:**
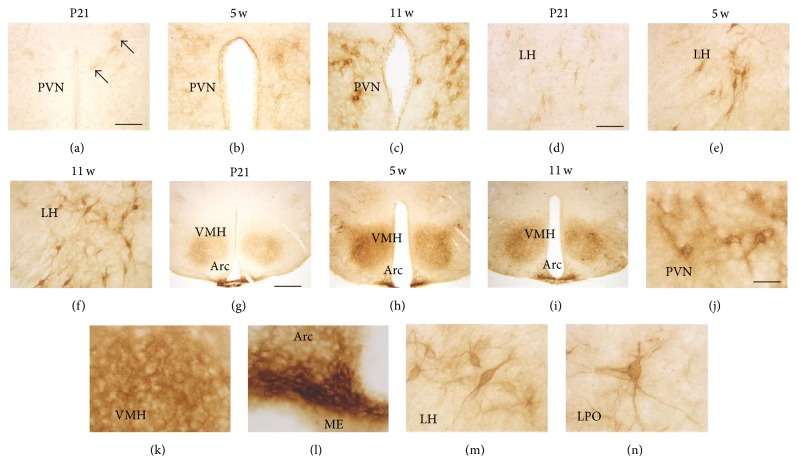
WFA-stained ECM in the developing hypothalamus. (a–i) WFA-labeled images in the PVN (a–c), LH (d–f), and VMH/Arc (g–i) at P21 (a, d, g), 5 w (b, e, h), and 11 w (c, f, i). Ambiguous PNN-like staining (arrows) was observed in the PVN at P21, which became gradually clear over 5 w to 11 w. PNN-like staining was observed at P21 in the LH, which became clearer at 5 w and 11 w. In all stages shown, WFA reactivity in the VMH was diffuse, while that of the Arc was dense, particularly in its ventral portion neighboring the ME. (j–n) Higher magnification images of WFA labeling at 11 w in the PVN (j), VMH (k), Arc (l), LH (M), and LPO (n). PNN structures in the PVN showed a diffuse manner, while those of the LH and LPO clearly surrounded cell bodies and dendrites. In the VMH and Arc, WFA reactivity did not surround dendrites. Arc: arcuate nucleus; LH: lateral hypothalamus; LPO: lateral preoptic area; ME: median eminence; PVN: paraventricular nucleus; VMH: ventromedial hypothalamic nucleus. Scale bars = 100 (a–f), 400 (g–i), and 40 (j–n) *μ*m.

In the hypothalamus, WFA labeling patterns and their developmental changes were different within each nucleus or area. In the paraventricular nucleus (PVN), especially in its anterior division, faint PNN-like labeling was observed at P21 (Figure 4(a)), which gradually became clear from 5 w (Figure 4(b)) to 11 w (Figure 4(c)). In the lateral hypothalamus (LH), a comparatively clear PNN-like form was observed at P21 (Figure 4(d)), which became clearer at 5 w (Figure 4(e)) and 11 w (Figure 4(f)). In the ventromedial hypothalamic nucleus (VMH), WFA reactivity was diffusely observed throughout the region at P21 (Figure 4(g)), which became stronger but remained diffuse at 5 w (Figure 4(h)) and 11 w (Figure 4(i)). Contrary to the VMH findings, strong WFA reactivity was observed in the ventral portion of the arcuate nucleus (Arc) neighboring the median eminence (ME) (Figures 4(g)–4(i)). High-power images at 11 w are represented in Figures 4(j)–4(n): PNNs in the PVN surrounded both cell bodies and dendrite-like processes, but they were somewhat diffuse compared with those of the LH (Figure 4(m)) and lateral preoptic area (LPO) (Figure 4(n)). WFA reactivity in the VMH was diffuse and weakly accumulated around particular cell bodies (Figure 4(k)). In the Arc, WFA reactivity densely surrounded cell bodies (Figure 4(l)).

**Figure 5:**
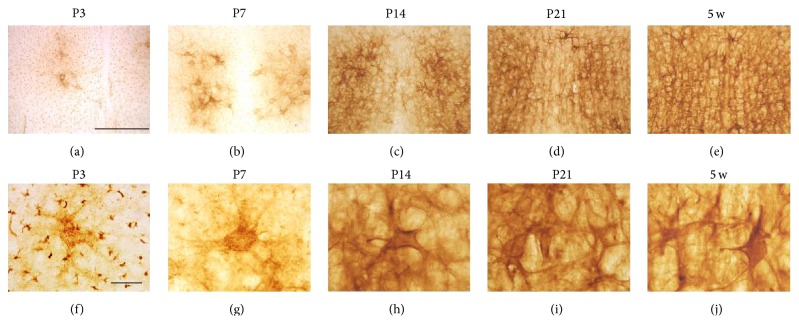
WFA-stained ECM in the Gi of the pons. (a–j) Low- (a–e) and high- (f–j) power images of WFA labeling in the Gi at P3 (a, f), P7 (b, g), P14 (c, h), P21 (d, i), and 5 w (e, j). PNN-like staining was observed at P3 and P7, which became clearer and more complicated from P14 onward. Gi: gigantocellular nucleus. Scale bars = 500 (a–e) and 50 (f–j) *μ*m.

In the gigantocellular nucleus of the pons (Gi), a few PNN structures surrounding both cell bodies and dendrites were observed at P3 (Figures 5(a) and 5(f)) and a substantial number of PNNs could be detected at P7 (Figures 5(b) and 5(g)). From P14 onward, WFA reactivity became stronger and appeared to make plexuses, in which individual PNNs were difficult to identify (Figures 5(c)–5(e) and 5(h)–5(j)).

**Figure 6:**
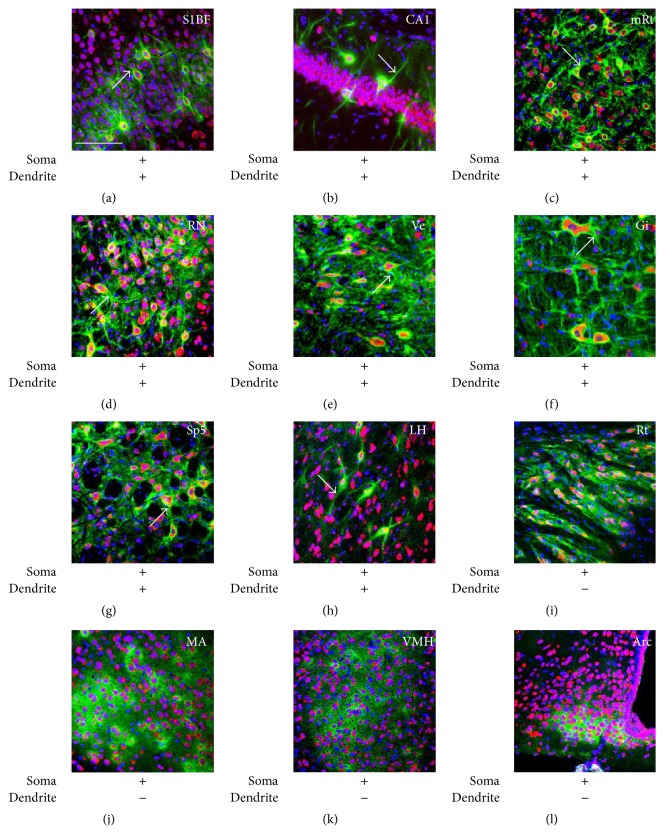
Structural variety of CSPG-contained ECM structures. (a–l) Fluorescent triple labeling of WFA (green), Nissl (red, neuron marker), and DAPI (blue, nucleus marker) at 11 w. Arrows indicate WFA signals around dendrites. The sign of plus (+) or minus (−) below images indicates the presence or absence of WFA reactivity around somata and dendrites. WFA signals in the Cx (a), hippocampal CA1 (b), mRt (c), RN (d), Ve (e), Gi (f), Sp5 (g), and LH (h) were observed around both somata and dendrites, while those of the Rt (i), MA (j), VMH (k), and Arc (l) did not show clear reactivity surrounding dendrites. Arc: arcuate nucleus; Gi: gigantocellular nucleus; LH: lateral hypothalamus; MA: medial amygdaloid nucleus; mRt: mesencephalic reticular formation; RN: red nucleus; Rt: reticular thalamic nucleus; S1BF: barrel field of the primary somatosensory cortex; Sp5: spinal trigeminal nucleus; Ve: vestibular nucleus; VMH: ventromedial hypothalamic nucleus. Scale bar = 100 *μ*m (a–l).

WFA-labeled ECM structures at 11 w were examined by fluorescent labeling with WFA, Nissl (neuron marker), and DAPI (nuclear marker). PNN-like WFA reactivity that was condensed around both cell bodies and dendrites was observed in many cortical, limbic, and brain stem areas, including the S1BF (Figure 6(a)), hippocampal CA1 (Figure 6(b)) and CA3 (data not shown), BLA (data not shown), lateral septum (data not shown), mesencephalic reticular formation (mRt, Figure 6(c)), red nucleus (RN, Figure 6(d)), vestibular nucleus (Ve, Figure 6(e)), Gi (Figure 6(f)), and spinal trigeminal nucleus (Sp5, Figure 6(g)). In the hypothalamus, the typical PNN structure was observed in the LH (Figure 6(h)) and LPO (data not shown). On the other hand, WFA reactivity in the reticular thalamic nucleus (Rt, Figure 6(i)), MA (Figure 6(j)), VMH (Figure 6(k)), and Arc (Figure 6(l)) was observed to surround cell bodies but not dendrites. WFA reactivity in the MA (Figure 6(j)) and VMH (Figure 6(k)) was particularly more diffuse and showed a more loosely organized accumulation around cell bodies. These results suggested structural variety of CSPG-contained ECM in the adult brain.

## 4. Discussion

The present study investigated developmental changes and adult structural variety of CSPG-contained ECM structures. Since the carbohydrate epitope of WFA is thought to be on aggrecan [21], the present results are probably related to the expression and localization of aggrecan. We first hypothesized that if the beginning of PNN formation implies the end of the critical period, the start of PNN formation should be different among brain regions, probably depending on their functions. Consistent with this hypothesis, our results showed a time difference with regard to the beginning of PNN formation.  Table 1 indicates the starting period of PNN formation in each brain region, together with known critical periods concerning various biological systems. Since particular regions marked with asterisks did not include typical PNN forms, indicated periods represent when WFA reactivity was first detected. Our results clearly showed that the earliest formation of PNNs occurred in some reticular formation nuclei by P3. Subsequent formation was observed in the primary sensory cortices and other nuclei of the brain stem by P7, and all regions began to form PNNs or express WFA-labeled CSPGs by P21. These findings suggest that the end of the critical period and the speed of brain maturation largely differ depending on regions.

Among the cerebral cortices, including the prefrontal, sensorimotor, and cingulate, immature PNNs were first observed in the S1, S1BF, and piriform cortex (Pir) and successively detected in the primary auditory cortex (Au1) and in the ventral and lateral orbital cortices (VO, LO) (Table 1). At P14, PNN formation began in nearly all regions, except for the V2MM. The critical period for whisker-barrel formation occurs by P7 [22], and formation of the barrel structure requires sensory inputs from whiskers [23], which are relayed to the Sp5 via the trigeminal nerve [24]. Interestingly, PNN formation in the Sp5 was also observed at P7 (Table 1). These results suggest cooperative maturation of the whisker sensory system. In the case of rodents, pups start to move independently by P10 [25]. Additionally, the critical period for the neuromuscular junction is P12 in mice [22]. Thus, our results seem to be consistent with the end of the critical period for the sensorimotor system.

In the hippocampus, PNN formation is observed at P14, except for in the dentate gyrus (Table 1). Furthermore, the notable feature in the hippocampus is dramatic maturation for 1 week from P14 to P21, while PNNs in other regions gradually mature, over approximately 2 weeks. Defining the critical period of the hippocampus is a very difficult task. However, the stress-hyporesponsive period (SHRP) may be related to the hippocampal critical period. The SHRP is the critical period for determining the future state of stress responses, including activity of the hypothalamic-pituitary-adrenal (HPA) axis [26, 27]. The hippocampus is a superordinate structure controlling HPA axis activity, which is also known as the hippocampal-HPA axis [28]. The SHRP in rodents approximately matches the first two postnatal weeks. Indeed, our previous study indicated that maternal separation performed during the first two postnatal weeks increases basal plasma corticosterone levels in adulthood, whereas the same intervention during the next week (i.e., from 3 w to 4 w) does not affect adult corticosterone levels [29–31]. Although it remains fully unknown whether PNN appearance in the hippocampus affects the end of the SHRP, this is a challenging issue that requires future study.

In the amygdala, PNN formation was observed by P21 (Table 1), which is mostly consistent with a previous study demonstrating a few obscure PNNs that were observed at P16 and that were found to dramatically increase by P21 [16]. In rodents, persistent fear memory can be observed in animals older than 3 w [32]. Similar to a previous study [16], our results support the functional importance of PNNs in the consolidation and maintenance of fear memory. Interestingly, WFA labeling showed that the MA had a loosely organized ECM structure that was diffuse in the neuropil and avoided surrounding dendrites. A previous study in the cerebellum termed a loosely organized ECM “semiorganized matrix” [33]. This term is thought to be applicable to the ECM structure of the MA. The MA is highly plastic throughout life and known as a sexually dimorphic region. As such, the density of dendritic spines in the MA is largely affected by sex steroids and actively changes during the female estrous cycles [34]. We suspect that the lack of CSPG matrix around MA neuron dendrites was related to the long-lasting maintenance of higher synaptic plasticity throughout adulthood.

Like the MA, many hypothalamic regions do not follow typical PNN structures. WFA labeling patterns observed in the VMH and Arc are also considered to be a semiorganized matrix [33]. Similar to the MA, hypothalamic neurons are ordinarily required to respond to lifelong hormonal and environmental changes for survival and species preservation. Hypothalamic neurons are generally thought to be plastic throughout adulthood [35–40], and the Arc and VMH are no exception because they control appetite and feeding behaviors in response to nutrient availability [35, 38, 41, 42]. Importantly, the structural and functional integrity of PNNs are maintained by complex interactions of multiple ECM molecules. In fact, even the lack of a single ECM molecule can affect both the structure and function of PNNs, as observed in knockout mice that lack genes encoding the hyaluronan proteoglycan link protein (HAPLN) 1 [43], HAPLN4 [44], and tenascin R [45]. Thus, our results remind us that the semiorganized CSPG matrix may not so much restrict synaptic plasticity as highly organized typical PNNs are thought to do [15, 46].

Among the brain regions examined, PNN appearance was earliest in the Gi and oral pontine reticular nucleus (PnO). The Gi is known to innervate the hypoglossal nucleus that controls movement of the tongue muscles [47]. The PnO is involved in the generation and maintenance of rapid eye movement (REM) sleep [48, 49]. These observations suggest that the formation of PNNs is dependent on neuronal activity [50–52], since pups are supposed to move their tongue muscles during suckling, and REM sleep is the most frequent type of sleep among newborns [53]. Furthermore, PNN formation begins by P7 in several regions of the brain stem, the RN, superior colliculus (SC), Sp5, and Ve. Major functions of these regions are as follows: SC, saccadic eye movements, oculosensory reflexes, and eye-head coordination; Sp5, sensory transmission from the face, including the whiskers; and Ve, the maintenance of equilibrium, posture, and the perception of head position. Importantly, the early beginnings of PNN formation in these regions suggest early maturation of nuclei related to the cranial nerves.

## 5. Conclusions

The present study systematically describes the development and structural variety of the brain ECM. These results strongly support the idea that PNN formation, as well as PNN structural integrity, indicates the degree of brain maturation. The present findings could be useful for determining critical periods among several brain regions.

## Supplementary Material

WFA staining was performed using sections pre-treated with ChABC to confirm the specificity of WFA binding. This treatment almost completely abolished WFA reactivity in the sections of the primary somatosensory cortex (S1, Fig. S1A), piriform cortex (Pir, Fig. S1B), reticular thalamic nucleus (Rt, Fig. S1C), hippocampus (Hc, Fig. S1D), ventromedial hypothalamic nucleus/arcuate nucleus (VMH/Arc, Fig. S1E), inferior colliculus (IC, Fig. S1F), and gigantocellular reticular nucleus (Gi, Fig. S1G) at 11w. However, small dot-like reactivity was observed in sections of the Hc at P7 after the same treatment (Fig. S1H). Such dot-like WFA reactivity was widely and frequently observed in immature brain sections and considered a false positive reaction of WFA.

## Figures and Tables

**Table 1:** The beginning periods of PNN formation in the developing brain. “†” indicates the periods that immature PNNs could be first detected. Asterisks (*∗*) indicate brain regions not having typical PNN forms and “‡” in these regions indicates the period that WFA signals were first detected. “¶” in the bottom rows shows known critical periods and their references in brackets.

	P3	P7	P9	P14	P21	5 W	11 W
Prefrontal cortex							
Frontal association (FrA)				†			
Orbital, medial (MO)				†			
Orbital, ventral and lateral (VO, LO)			†				
Prelimbic (PrL)				†			
Sensorimotor cortex							
Auditory, primary (Au1)			†				
Motor, primary (M1)				†			
Motor, secondary (M2)				†			
Piriform cortex (Pir)		†					
Somatosensory, primary (S1)		†		†			
Somatosensory, primary, barrel field (S1BF)		†		†			
Somatosensory, secondary (S2)				†			
Visual, primary (V1)				†			
Visual, secondary, and lateral (V2L)				†			
Visual, secondary, and mediolateral (V2ML)				†			
Visual, secondary, and mediomedial (V2MM)					†		
Cingulate gyrus							
Anterior cingulate (Cg)				†			
Posterior cingulate (retrosplenial dysgranular) (RSG)				†			
Basal ganglia							
Caudate putamen (CPu)				†			
Ventral pallidum (VP)					†		
Thalamus							
Habenular nucleus, lateral (LHb)				†			
Reticular thalamic nucleus (Rt)^*∗*^				‡			
Zona incerta (ZI)				†			
Hypothalamus							
Arcuate nucleus^*∗*^			‡				
Lateral hypothalamus (LH)				†			
Lateral preoptic area (LPO)					†		
Lateral mammillary nucleus (LM)				†			
Ventromedial hypothalamic nucleus (VMH)^*∗*^			‡				
Paraventricular nucleus, anterior (aPVN)					†		
Limbic system							
Amygdala, basolateral (BLA)					†		
Amygdala, central (Ce)					†		
Amygdala, medial (MA)^*∗*^					‡		
Bed nucleus of the stria terminalis (BNST)					†		
Hippocampus, CA1 (CA1)				†			
Hippocampus, CA2 (CA2)^*∗*^				‡			
Hippocampus, CA3 (CA3)				†			
Hippocampus, dentate gyrus (DG)					†		
Septum, lateral (LS)					†		
Septum, medial (MS)					†		
Brain stem							
Anterior pretectal nucleus (APT)				†			
Gigantocellular reticular nucleus (Gi)	†						
Inferior colliculus (IC)				†			
Interstitial nucleus of Cajal (InC)				†			
Mesencephalic reticular formation (mRt)				†			
Paratrochlear nucleus (Pa4)			†				
Pontine reticular nucleus, oral (PnO)	†						
Precuneiform area (prCnF)				†			
Red nucleus (RN)		†					
Substantia nigra (SN)				†			
Superior colliculus		†					
Spinal trigeminal nucleus (Sp5)		†					
Ventral tegmental area (VTA)					†		
Vestibular nuclei (Ve)		†					
Critical period							
Whisker-barrel formation [22]		<P7^¶^					
Neuromuscular junction [22]			<P12^¶^				
Stress-hyporesponsive period (HPA axis) [26, 27]				<P14^¶^			
Stress/anxiety [22]					<P21^¶^		
Erasable fear memory [32]					<P21^¶^		
Orientation bias [22]					<4 w^¶^		
Ocular dominance [22]						<5 w^¶^	

## Abbreviations

Arc: Arcuate nucleus
Au1: Primary auditory cortex
BLA: Basolateral amygdaloid nucleus
Cg: Cingulate cortex
ChABC: Chondroitinase ABC
CS: Chondroitin sulfates
CSPGs: Chondroitin sulfate proteoglycans
ECM: Extracellular matrix
Gi: Gigantocellular reticular nucleus
HAPLN: Hyaluronan proteoglycan link protein
HPA axis: Hypothalamic-pituitary-adrenal axis
InC: Interstitial nucleus of Cajal
LH: Lateral hypothalamus
LO: Lateral orbital cortex
LPO: Lateral preoptic area
M1: Primary motor cortex
M2: Secondary motor cortex
MA: Medial amygdaloid nucleus
ME: Median eminence
mRt: Mesencephalic reticular formation
P: Postnatal day
PBS: Phosphate-buffered saline
PBST: PBS containing 0.3% Triton X-100
Pir: Piriform cortex
PNNs: Perineuronal nets
PnO: Oral pontine reticular nucleus
PrL: Prelimbic cortex
PVN: Paraventricular nucleus
RN: Red nucleus
RSG: Retrosplenial granular cortex
Rt: Reticular thalamic nucleus
S1: Primary somatosensory cortex
S1BF: The barrel field of the primary somatosensory cortex
SC: Superior colliculus
SHRP: Stress-hyporesponsive period
SNR: Reticular part of the substantia nigra
Sp5: Spinal trigeminal nucleus
V1: Primary visual cortex
V2L: Lateral area of the secondary visual cortex
V2M: Medial area of the secondary visual cortex
Ve: Vestibular nucleus
VMH: Ventromedial hypothalamic nucleus
VO: Ventral orbital cortex
w: Postnatal week
WFA: * Wisteria floribunda* agglutinin.
