# Supplementary material for: Development and Structural Variety of the Chondroitin Sulfate Proteoglycans-Contained Extracellular Matrix in the Mouse Brain
Source: Neural Plast. 2015 Nov 16;2015:256389. doi: 10.1155/2015/256389 (PMC4663360; doi:10.1155/2015/256389)
Supplement: Supplementary file 1 — WFA staining was performed using sections pre-treated with ChABC to confirm the specificity of WFA binding. This treatment almost completely abolished WFA reactivity in the sections of the primary somatosensory cortex (S1, Fig. S1A), piriform cortex (Pir, Fig. S1B), reticular thalamic nucleus (Rt, Fig. S1C), hippocampus (Hc, Fig. S1D), ventromedial hypothalamic nucleus/arcuate nucleus (VMH/Arc, Fig. S1E), inferior colliculus (IC, Fig. S1F), and gigantocellular reticular nucleus (Gi, Fig. S1G) at 11w. However, small dot-like reactivity was observed in sections of the Hc at P7 after the same treatment (Fig. S1H). Such dot-like WFA reactivity was widely and frequently observed in immature brain sections and considered a false positive reaction of WFA. [file 256389.f1.pdf]

Supplementary information

Figure S1. Confirmation of WFA specificity for CS by treating sections with ChABC. (A-H) WFA–labeled images after treatment without (-) (left column) and with (+) (right column) ChABC. Pictures of (A-G) and (H) were from sections at 11w and P7, respectively. Note that WFA reactivity was almost completely abolished after ChABC treatment at 11w (A-G), while small dot-like reactivity (arrowheads, H) had not disappeared following the same treatment. Arc, arcuate nucleus; ChABC, chondroitinase ABC; Gi, gigantocellular nucleus; Hc, hippocampus; IC, inferior colliculus; Pir, piriform cortex; Rt, reticular thalamic nucleus; S1, primary somatosensory cortex; VMH, ventromedial hypothalamic nucleus. Scale bars = 500 (A-G) and 250 (H) μm.

Fig. S1

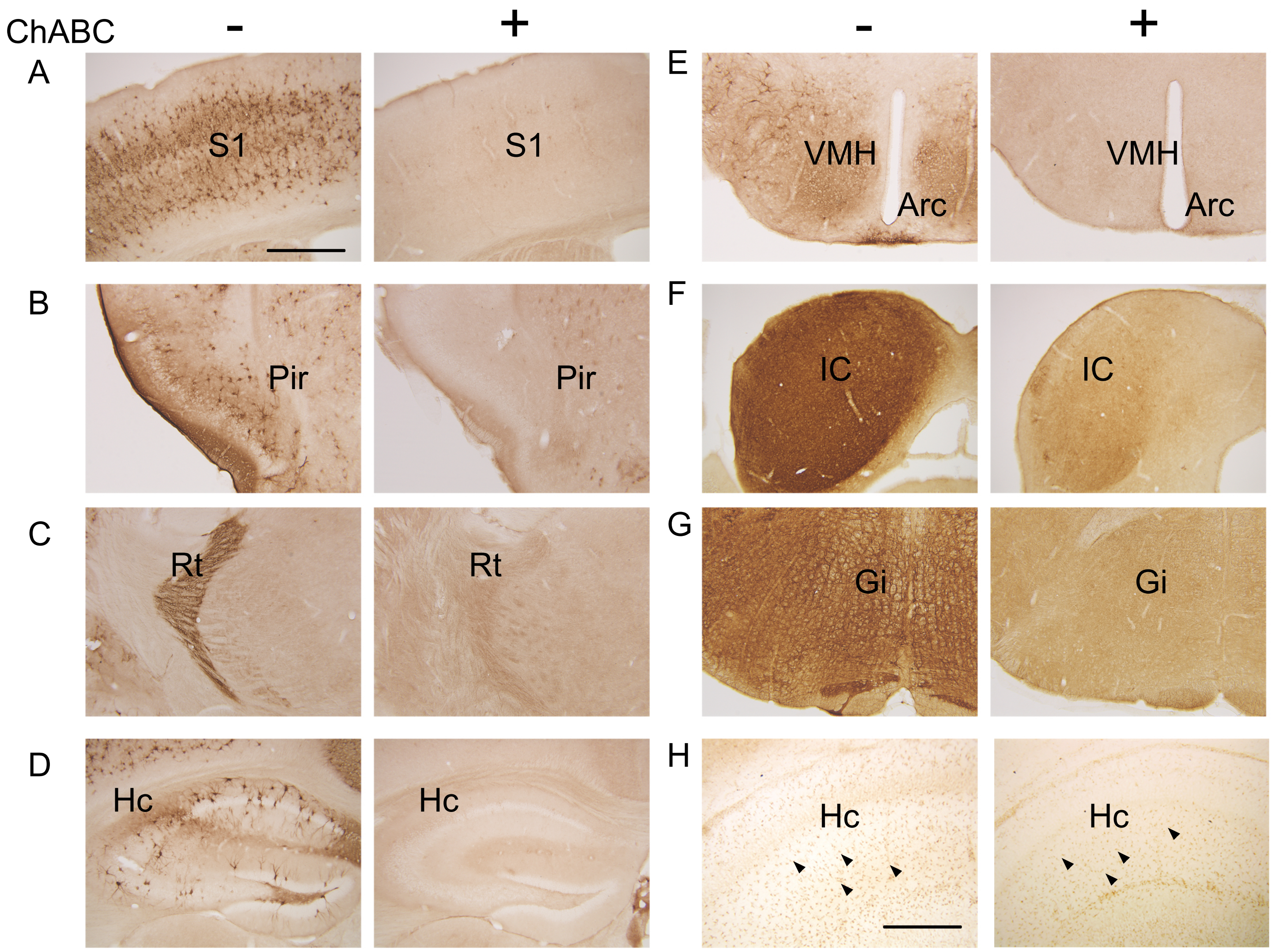
ChABC
-
+
-
+
A
S1
S1
E
VMH
Arc
VMH
Arc
B
Pir
Pir
F
IC
IC
C
Rt
Rt
G
Gi
Gi
D
Hc
Hc
H
Hc
Hc
